# *In Vitro* Studies on Erythrosine-Based Photodynamic Therapy of Malignant and Pre-Malignant Oral Epithelial Cells

**DOI:** 10.1371/journal.pone.0034475

**Published:** 2012-04-02

**Authors:** Abhishek D. Garg, Muthiah Bose, Mohammed I. Ahmed, William A. Bonass, Simon R. Wood

**Affiliations:** 1 Department of Oral Biology, Leeds Dental Institute, Faculty of Medicine and Health, University of Leeds, Leeds, United Kingdom; 2 Faculty of Biological Sciences, University of Leeds, Leeds, United Kingdom; Ospedale Pediatrico Bambino Gesù, Italy

## Abstract

Photodynamic Therapy (PDT) involves the administration of a tumor localizing photosensitizing agent, which upon activation with light of an appropriate wavelength leads to the destruction of the tumor cells. The aim of the present study was to determine the efficacy of erythrosine as a photosensitizer for the PDT of oral malignancies. The drug uptake kinetics of erythrosine in malignant (H357) and pre-malignant (DOK) oral epithelial cells and their susceptibility to erythrosine-based PDT was studied along with the determination of the subcellular localization of erythrosine. This was followed by initial investigations into the mechanism of cell killing induced following PDT involving both high and low concentrations of erythrosine. The results showed that at 37°C the uptake of erythrosine by both DOK and H357 cells increased in an erythrosine dose dependent manner. However, the percentage of cell killing observed following PDT differed between the 2 cell lines; a maximum of ∼80% of DOK cell killing was achieved as compared to ∼60% killing for H357 cells. Both the DOK and H357 cell types exhibited predominantly mitochondrial accumulation of erythrosine, but the mitochondrial trans-membrane potential (ΔΨ_m_) studies showed that the H357 cells were far more resistant to the changes in ΔΨ_m_ when compared to the DOK cells and this might be a factor in the apparent relative resistance of the H357 cells to PDT. Finally, cell death morphology and caspase activity analysis studies demonstrated the occurrence of extensive necrosis with high dose PDT in DOK cells, whereas apoptosis was observed at lower doses of PDT for both cell lines. For H357 cells, high dose PDT produced both apoptotic as well as necrotic responses. This is the first instance of erythrosine-based PDT's usage for cancer cell killing.

## Introduction

Oral cancer is one of the most disfiguring and debilitating malignancies [1] and an important problem especially in those countries where tobacco chewing or smoking is prevalent [2]. In 2002, oral cancers accounted for approximately 274,000 cases world-wide [3]. Surgery, radiation therapy and chemotherapy are the typical traditional methods used for oral cancer treatment. Although these traditional oral cancer treatment strategies have advanced several fold over last few decades (*e.g.* discovery of new chemotherapeutics, powerful imaging tools allowing for better diagnostics-driven surgery and fluorescence/bioluminescence-guided surgery), the 5-year survival rate for oral cancer victims hasn't improved significantly over the past 50 years [4]. In addition to this, these treatment strategies have several mild to severe side-effects *e.g.* salivary gland dysfunction, mucositis/stomatitis, dental caries/demineralization, neurotoxicity and osteoradionecrosis [1], [4]. Thus, an alternative treatment-strategy is desirable which could be utilized for treating tumours in the oral cavity in a simple, efficient, cost-effective and patient-friendly manner [4]. One such emerging treatment strategy is Photodynamic Therapy (PDT) [5], [6], [7].

The principle of PDT is that an administered photosensitizer (PS) is selectively retained by cancer cells [7] and when the tumour is irradiated with light of the appropriate wavelength, the PS is activated and causes tumour cell death by the production of reactive oxygen species (ROS) and free radicals [7], [8], [9].

The main cancer-types for which PDT has been widely applied include malignancies of head and neck, brain, lung, prostate, ovaries, skin and oesophagus [6], [10], [11], [12]. In the past, PDT has been applied to oral cancer using various photosensitizers, *e.g.*, dihaematoporphyrin, meta tetrahydroxy phenyl chlorine (mTHPC) or Foscan and 5-aminolaevulinic acid [13]. Though PDT treatments for oral cancer using the above photosensitizers are effective there are some limitations [14], [15] including the lengthy clinical and legislative assessments that significantly delay translation of many such photosensitizers into the clinic. For instance, while EU has approved usage of Foscan-based PDT for treatment of early head and neck cancers; FDA has not yet approved PDT treatment for head and neck squamous cell carcinoma [4]. Such scenarios, have encouraged the search for other photosensitizers with more immediate benefits and possibilities of fast clinical translation [15]. To this end, our research has utilized the photosensitizer, Erythrosine B. Three major advantages of erythrosine over certain other photosensitizers are – (1) it is not toxic to the host, (2) it is approved by the FDA for usage in food/food products and (3) it has already been approved for use in dentistry, thereby making its translation into clinic easier (in case of promising pre-clinical data) than possible for other photosensitizers, as far as oral cancer is concerned [16], [17].

The potential of erythrosine to undergo photochemical reactions and generate singlet oxygen was known previously [18] and it has been used by ourselves as a photosensitizer of bio-films of *Streptococcus mutans* for the PDT-based cell killing of these oral infectious bacteria [17]. This suggested that erythrosine may also be an effective photosensitizing agent for the treatment of cancer cells [19].

The efficacy of PDT is related to a number of factors which include the cellular uptake kinetics of the photosensitizer and its subcellular localization characteristics [5], [7], [9]. Since erythrosine is a hydrophilic dye [20], it is likely to accumulate preferentially in lysosomes and mitochondria [21]. These localization characteristics may help in determining the mechanism by which cell death occurs following PDT.

The site of localization of a PS is usually the primary target for PDT since the ROS produced due to the PDT have a very short life-time (<0.05 µs) [5], [22], [23]. Upon activation using the appropriate wavelength of light, the PS absorbs the light photon energy (h*v) and ascends from ground-state to the excited singlet state resulting in the production of ROS [7], [24]. ROS production due to PDT causes a highly elevated oxidative stress in the cell, the magnitude of which depends upon the PDT dose applied and which also governs the pathway of cell-death following PDT [6], [14], [24], [25]. The two most well-characterized cell death mechanisms following PDT are apoptosis and (primary) necrosis [5], [6], [25]. Necrosis usually predominates when using high dose PDT whereas apoptosis is more usually seen with comparatively lower PDT doses.

Primary necrosis is principally a passive cell death process which occurs due to extreme external physical stress or severe intracellular damage, and is irreversible [21], [26].

Apoptosis (programmed cell death), on the other hand, is an active pathway of cell death which involves the activation of various cellular factors including initiator caspases (Caspase 2, 8, 9 and 10) and executioner caspases (Caspase 3 and 7) [27]. Apoptotic pathways (intrinsic and extrinsic) usually involve mitochondria, which is frequently regarded as the central processing organelle for apoptosis [25], [28]. Therefore, mitochondrial damage is often essential for the induction of apoptosis within cells following PDT [7], [25].

The aim of the present investigation was to determine the efficacy of erythrosine in the photodynamic therapy of oral malignancies. To this end, we have studied the uptake kinetics of erythrosine in malignant (H357) and pre-malignant (DOK) oral epithelial cells and their susceptibility to erythrosine-based PDT. Determination of the subcellular localization of erythrosine was followed by initial investigations into the mechanism of cell killing induced at both high and low concentrations of erythrosine.

## Results

### Erythrosine Uptake Analysis

**Figure 1:**
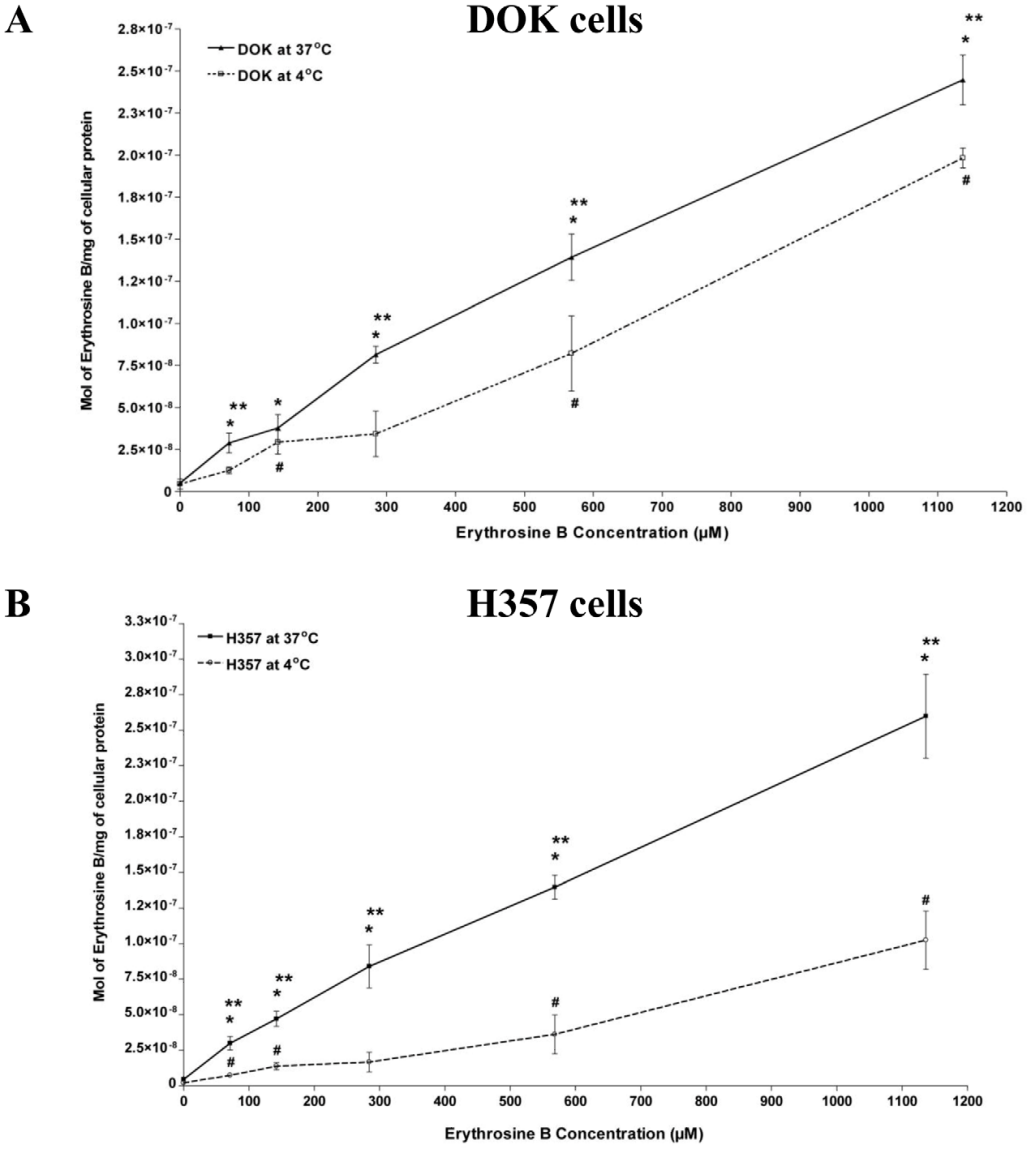
Erythrosine Uptake Analysis. Uptake of erythrosine by pre-malignant (DOK) and malignant (H357) oral epithelial cells was analyzed at 37°C and 4°C. (**A**) Drug uptake kinetics of erythrosine in DOK cells at 37°C and 4°C. (**B**) Drug uptake kinetics of erythrosine in H357 cells at 37°C and 4°C. The values are means of four replicate determinations ± S.E.M. Statistical analysis was done *via* student's t-test with significance set at P<0.05 (* indicates statistical comparison of 37°C data-points vs. the respective data values of 0 µM erythrosine; # indicates comparison of 4°C data-points vs. the respective data values of 0 µM erythrosine and ** indicates comparison between 37°C data-points and the corresponding 4°C data-points).

At 37°C for erythrosine concentrations ranging from 71.03 µM up to 1136.5 µM, the uptake of erythrosine by DOK cells was seen to increase in an erythrosine dose-dependent manner, such that it was significantly more (*P<0.05) for all the erythrosine concentrations than the 0 µM background (Figure 1A). A similar uptake pattern was seen for the H357 cells for erythrosine concentrations ranging from 71.03 µM to 1136.5 µM (Figure 1B). Statistical analysis showed that there was no significant difference in the uptake of erythrosine by the two cell lines at this temperature.

At 4°C the erythrosine uptake was considerably slower, especially at erythrosine concentrations from 71.03 µM up to 568.27 µM (Figure 1A and 1B) for both cell lines. Moreover for both cell lines (Figure 1A and 1B) at almost all erythrosine concentrations, the erythrosine uptake at 37°C was significantly higher than at 4°C (**P<0.05). However, the overall erythrosine uptake at 4°C was higher for DOK cells (Figure 1A) when compared to H357 cells (Figure 1B). Similarly, at 1136.5 µM the erythrosine uptake was significantly higher in DOK cells than in H357 cells.

### PDT based Cell Killing

**Figure 2:**
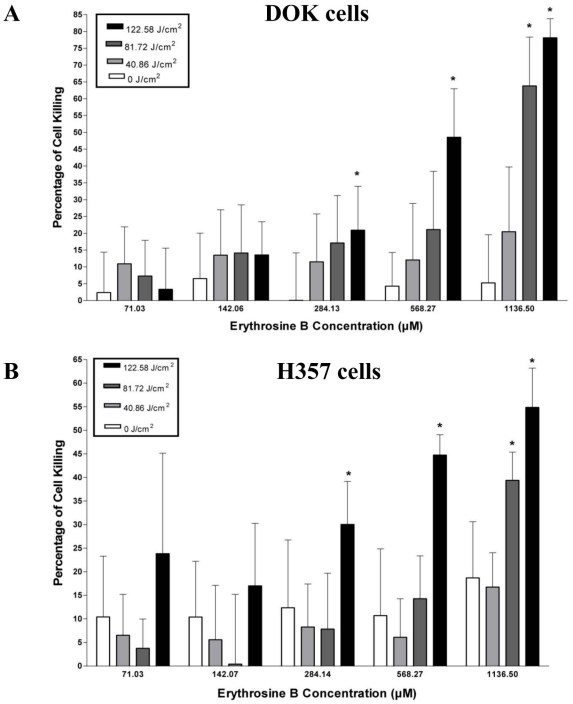
Cell Killing Assay. The pre-malignant (DOK) and malignant (H357) oral epithelial cells were incubated with different concentrations of erythrosine B (71.03 µM, 142.06 µM, 284.13 µM, 568.27 µM and 1136.50 µM) followed by PDT irradiation with different fluences (0 J/cm^2^, 40.86 J/cm^2^, 81.72 J/cm^2^ and 122.58 J/cm^2^). Following PDT, the cells were recovered 24 h later and percentage (%) of cell killing caused by PDT was estimated. (**A**) DOK cell's susceptibility to erythrosine-based PDT and (**B**) H357 cell's susceptibility to erythrosine-based PDT. The values are means of six replicate determinations ± S.D. Statistical analysis was done *via* student's t-test with significance set at P<0.05 (* indicates statistical comparison of a data-point within one erythrosine B concentration group vs. the corresponding data values of 0 J/cm^2^ fluencies for that group).

The overall percentage (%) cell killing caused by PDT in DOK cells rose in a light/fluence and photosensitizer dose-dependent manner (Figure 2A). Low PDT doses (all irradiations for erythrosine concentrations up to 284.13 µM as well as irradiation of 40.86 J/cm^2^ for erythrosine concentrations of 568.27 µM and 1136.5 µM) caused less cell kill which was not significantly higher than cell killing observed at same erythrosine concentrations without irradiation (*i.e.* fluence – 0 J/cm^2^). However, at higher PDT doses (irradiation fluence of 81.72 J/cm^2^ and/or 122.58 J/cm^2^ and erythrosine concentrations of 568.27 µM and/or 1136.5 µM) the observed cell kill rose rapidly such that it was significantly more (*P<0.05) than cell killing observed at same erythrosine concentrations without irradiation (*i.e.* fluence – 0 J/cm^2^). The maximum percentage cell kill was observed at 1136.5 µM concentration and 122.58 J/cm^2^ irradiation fluence (Figure 2A). The other dose showing appreciable cell killing was the 1136.5 µM concentration with 81.72 J/cm^2^ irradiation fluence. Overall, the LD_50_ for DOK cells was found to be 585.32 µM erythrosine at 122.58 J/cm^2^ irradiation fluence and 920.60 µM erythrosine at 81.72 J/cm^2^ irradiation fluence.

Cell killing trends following erythrosine-PDT were similar in the H357 cells (Figure 2B) as described above for DOK cells. However, cell killing caused by PDT in H357 cells was lower than in the DOK cells (Figure 2A and 2B). Maximum cell killing was observed at 1136.5 µM concentration and 122.58 J/cm^2^ irradiation fluence (Figure 2B). Overall, the LD_50_ for PDT-treated H357 cells was found to be 818.31 µM at 122.58 J/cm^2^ irradiation fluence. The LD_50_ was not reached for the other irradiation times in H357 cells. Lastly, for erythrosine concentrations above 1136.5 µM (*i.e.* 2.27–9.09 mM), increasingly significant cytotoxicity without irradiation (dark cytotoxicity of the photosensitizer; data not shown) was observed; hence in the present study the PDT-analysis has not been performed beyond the erythrosine concentration of 1136.5 µM. Thus the therapeutically relevant erythrosine concentration range for PDT was less than 1136.5 µM.

### Sub-cellular Localization Analysis by CLSM

**Figure 3:**
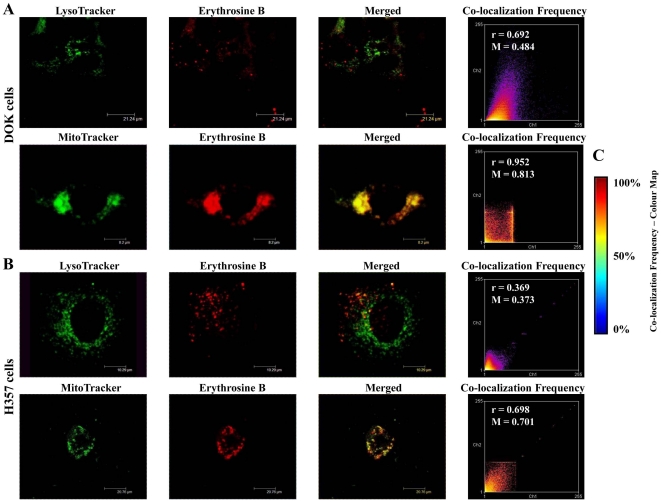
Erythrosine co-localization analysis. Sub-cellular co-localization of erythrosine in both DOK and H357 cells. (**A and B**) LysoTracker localization (for lysosomal visualization) and MitoTracker localization (for mitochondrial visualization) was compared with 1136.50 µM erythrosine localization in both DOK (**A**) as well as H357 (**B**) cells. Also, co-localization frequency scatter plots, Pearson's coefficient (r) and Mander's coefficient (M) were calculated to evaluate the overlap between LysoTracker/MitoTracker localizations and erythrosine localizations. (**C**) Co-localization frequency colour map indicates the estimation of co-localizations on the basis of ‘heat map schemes’ such that hotter colours tend to indicate high co-localization. Thus, red/maroon indicates 100% co-localization, yellow-green indicates 50% co-localization and purple/dark blue indicates 0% co-localization.

Co-localization analysis was carried out for erythrosine with respect to dyes that stain either lysosomes (LysoTracker) or mitochondria (MitoTracker). Apart from analysis of merged channels images, co-localization was also estimated *via* other (statistical) parameters like co-localization frequency scatter plots (based on co-localization frequency colour ‘heat’ maps), Pearson's coefficient and Mander's coefficient, as described elsewhere [29]. In case of both DOK (Figure 3A) and H357 (Figure 3B) cells, erythrosine was found to co-localize more strongly with MitoTracker than with LysoTracker as indicated by the following four parameters – (1) higher incidence of yellow co-localization colour in the merged channels image for MitoTracker/erythrosine, (2) hotter colours in the co-localization frequency scatter plot (tending towards 100% co-localization as per the colour map in Figure 3C) for MitoTracker/erythrosine, (3) higher Pearson's coefficient values for MitoTracker/erythrosine (r values, as indicated in the respective scatter plots) and (4) higher Mander's coefficient for MitoTracker/erythrosine (M values, as indicated in the respective scatter plots).

Based on this data, we can conclude that erythrosine localizes predominantly in the mitochondria in both the DOK as well as H357 cell lines.

### Erythrosine-PDT affects Mitochondrial Trans-membrane Potential (ΔΨ_m_) in DOK and H357 cells

**Figure 4:**
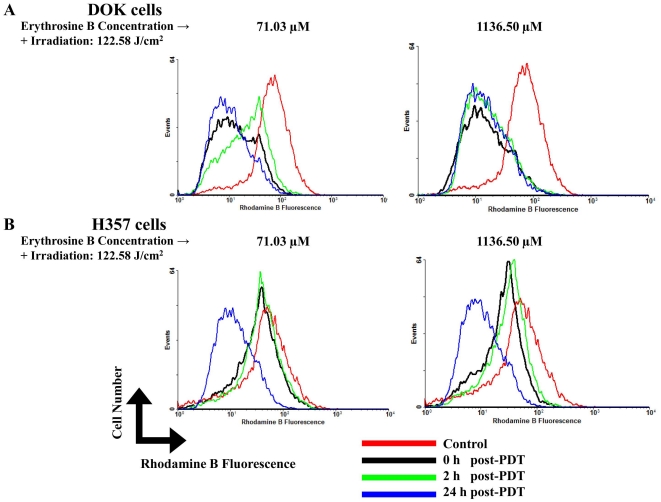
Mitochondrial trans-membrane potential analysis. Effects of PDT on mitochondrial trans-membrane potential (ΔΨ_m_) at different recovery time points post-PDT (0, 2 and 24 h) on DOK and H357 cells were analyzed at both low (71.03 µM) and high (1136.50 µM) doses of erythrosine. For both doses, PDT irradiation was done with a fluence of 122.58 J/cm^2^. **A,** ΔΨ_m_ studies on DOK cells at low (71.03 µM–122.58 J/cm^2^) and high (1136.50 µM–122.58 J/cm^2^) doses of erythrosine-PDT. **B,** ΔΨ_m_ studies on H357 cells at low (71.03 µM–122.58 J/cm^2^) and high (1136.50 µM–122.58 J/cm^2^) doses of erythrosine-PDT. The presented histograms are representative of three replicate determinations.

DOK and H357 cells treated with PDT using a low dose of erythrosine-PDT (71.03 µM erythrosine concentration and 122.58 J/cm^2^ irradiation fluence) showed different responses to the treatment in terms of changes in the ΔΨ_m_. Following low dose treatment, DOK cells demonstrated a considerable decrease in their overall ΔΨ_m_ immediately following PDT (Figure 4A). Interestingly, at 2 hrs recovery time, a specific population of cells with highly depolarized mitochondria shifted towards normalcy. However, at 24 hrs, the ΔΨ_m_ had collapsed further (Figure 4A).

Low dose-treated H357 cells showed no appreciable loss of their overall ΔΨ_m_ at the earlier recover time-points following PDT (Figure 4B). However, similar to the DOK cells (Figure 4A), at 24 hrs the ΔΨ_m_ suddenly collapsed drastically (Figure 4B).

Similarly, DOK and H357 cells treated with high dose (1136.5 µM–122.58 J/cm^2^) also showed different responses initially. While DOK cells immediately lost their ΔΨ_m_ after PDT (Figure 4A), the H357 cells initially resisted the change in ΔΨ_m_ at the earlier recovery time-points after PDT (Figure 4B). However, at 24 hrs after PDT, the ΔΨ_m_ collapsed drastically for both cell lines thereby pointing towards severe mitochondrial outer-membrane permeabilization (MMP).

### Cell Death Morphology analysis using CLSM and SEM

**Figure 5:**
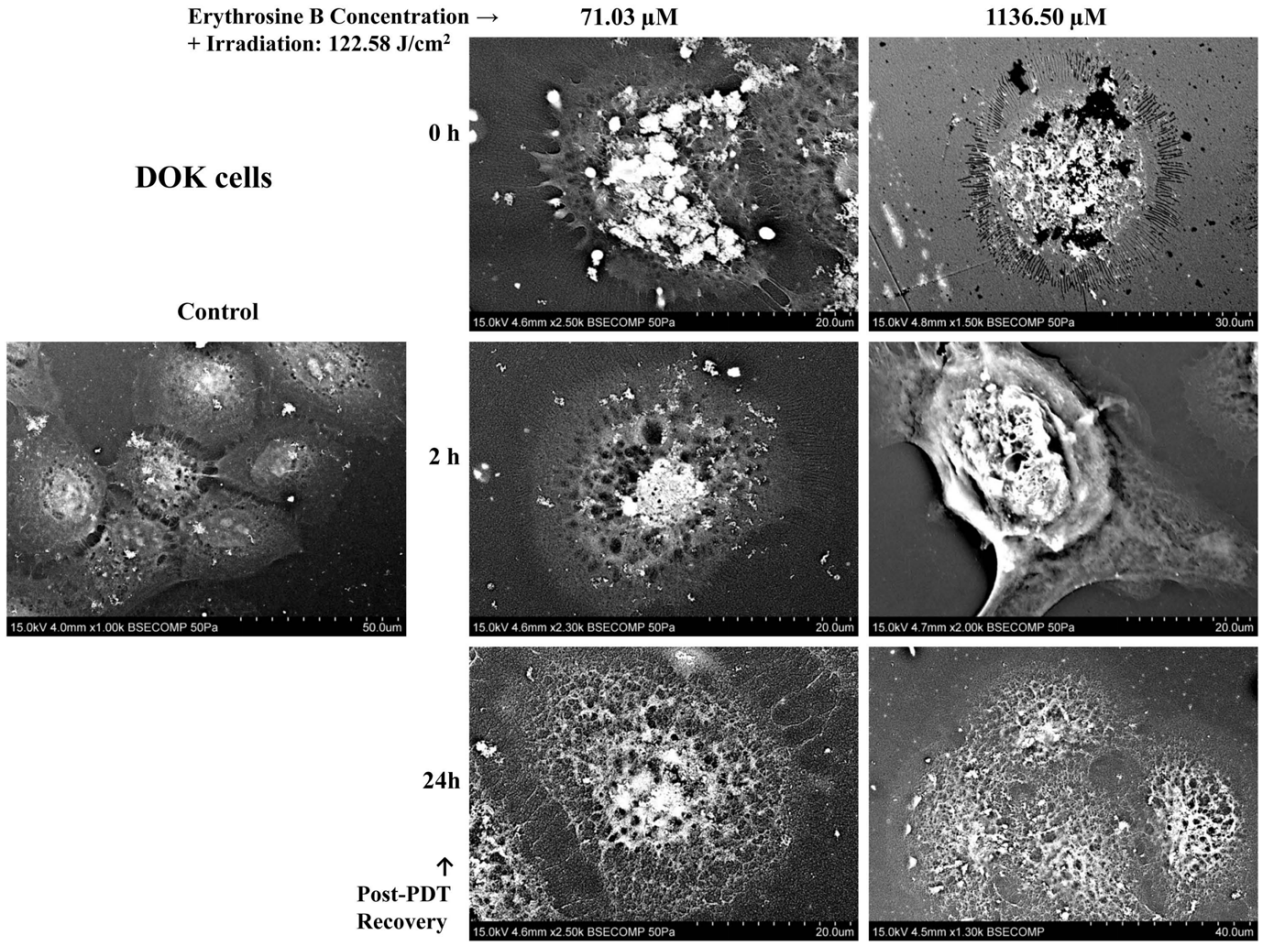
Scanning Electron Microscopy (SEM) Analysis on DOK cells. SEM images showing DOK cell morphologies at different recovery time points post-PDT (0, 2 and 24 h) were taken for both low (71.03 µM) and high (1136.50 µM) doses of erythrosine. For both doses, PDT irradiation was done with a fluence of 122.58 J/cm^2^. These images demonstrate the occurrence of apoptosis (and secondary necrosis) following PDT with low dose erythrosine and necrosis in the high dose condition.

**Figure 6:**
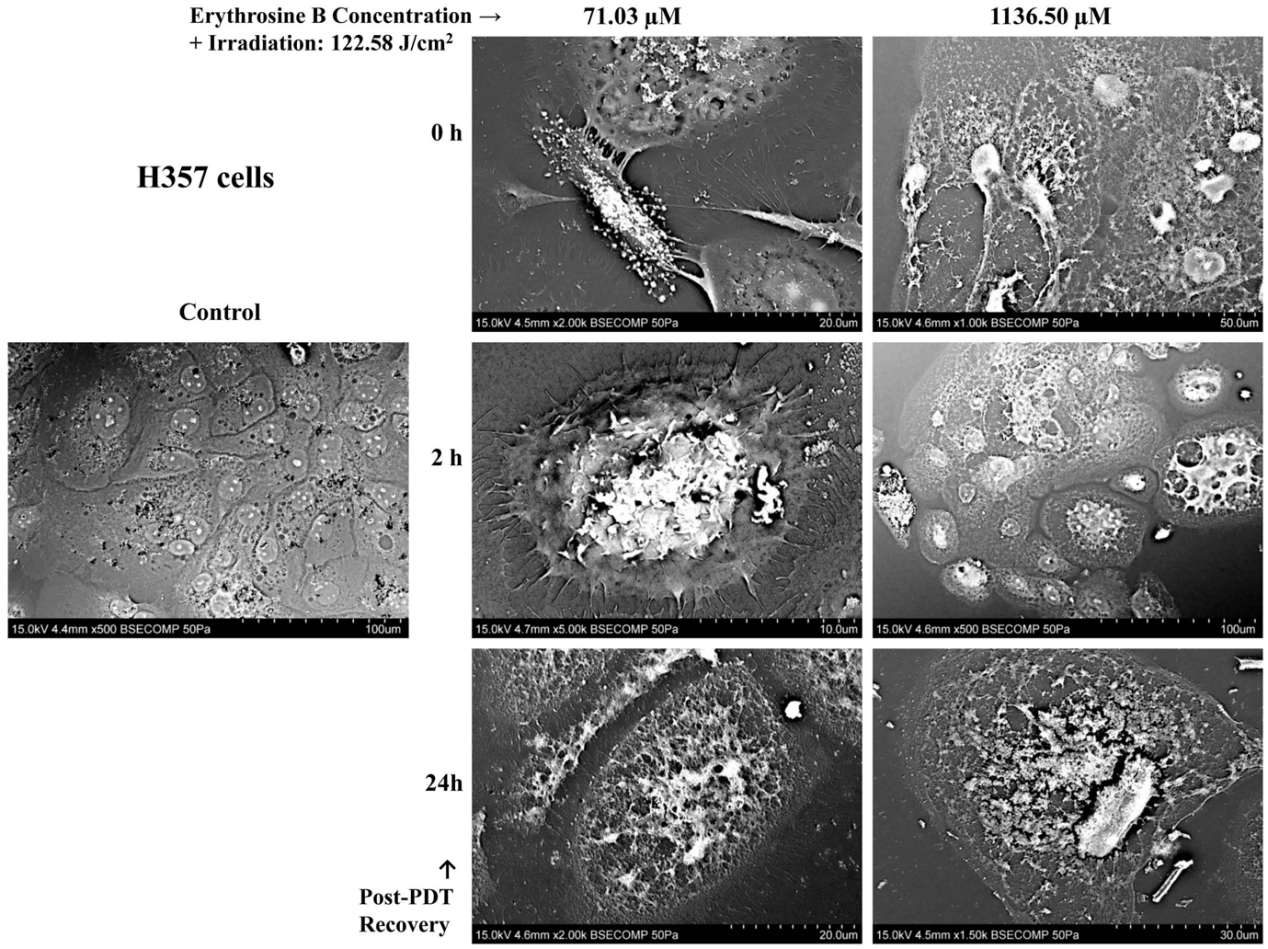
Scanning Electron Microscopy (SEM) Analysis on H357 cells. SEM images showing H357 cell morphologies at different recovery time points post-PDT (0, 2 and 24 h) were taken for both low (71.03 µM) and high (1136.50 µM) doses of erythrosine. For both doses, PDT irradiation was done with a fluence of 122.58 J/cm^2^. These images demonstrate the occurrence of apoptosis (and secondary necrosis) following PDT with low dose erythrosine and necrosis in the high dose condition.

**Figure 7:**
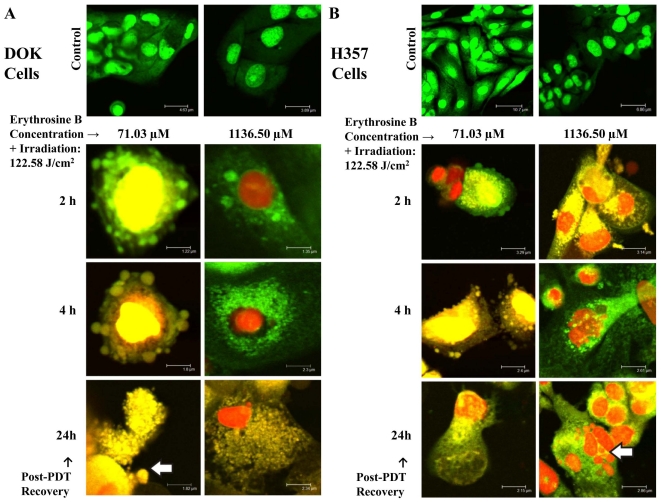
Confocal Laser Scanning Microscopy (CLSM) Analysis. CLSM images of DOK (**A**) and H357 (**B**) cells at different recovery time points post-PDT (2, 4 and 24 h) were taken for both low (71.03 µM) and high (1136.50 µM) doses of erythrosine. For both doses, PDT irradiation was done with a fluence of 122.58 J/cm^2^. Green (SYTO9) fluorescence represents both live and dead cells while red (Propidium Iodide) fluorescence stains the dead cells alone. The arrow in the DOK cells (**A**) indicates the presence of apoptotic bodies whereas the arrow in the H357 cells (**B**) points to a group of necrotic cells.

Untreated (Control) DOK and H357 cells showed a normal morphology in both the SEM (Figure 5 and 6) and CLSM analyses (Figure 7). Following PDT with low dose erythrosine-PDT (71.03 µM–122.58 J/cm^2^), we observed apoptotic-like characteristics such as membrane blebbing, cell shrinkage, loss of extensive cell-cell linkage and formation of apoptotic bodies at early recovery time points post-PDT (0–4 h) followed by secondary necrosis at 24 h post-PDT; in both DOK (Figure 5 and 7) and H357 cells (Figure 6 and 7). Following PDT with high dose erythrosine-PDT (1136.5 µM–122.58 J/cm^2^) we observed prominent necrotic characteristics such as extensive membrane and cell disintegration, extensive karyolysis and the presence of large amounts of cellular debris at all recovery time point post-PDT in DOK cells (Figure 5 and 7) along with some apoptotic characteristics in H357 cells (Figure 6 and 7).

### Caspase Activity Assay

**Figure 8:**
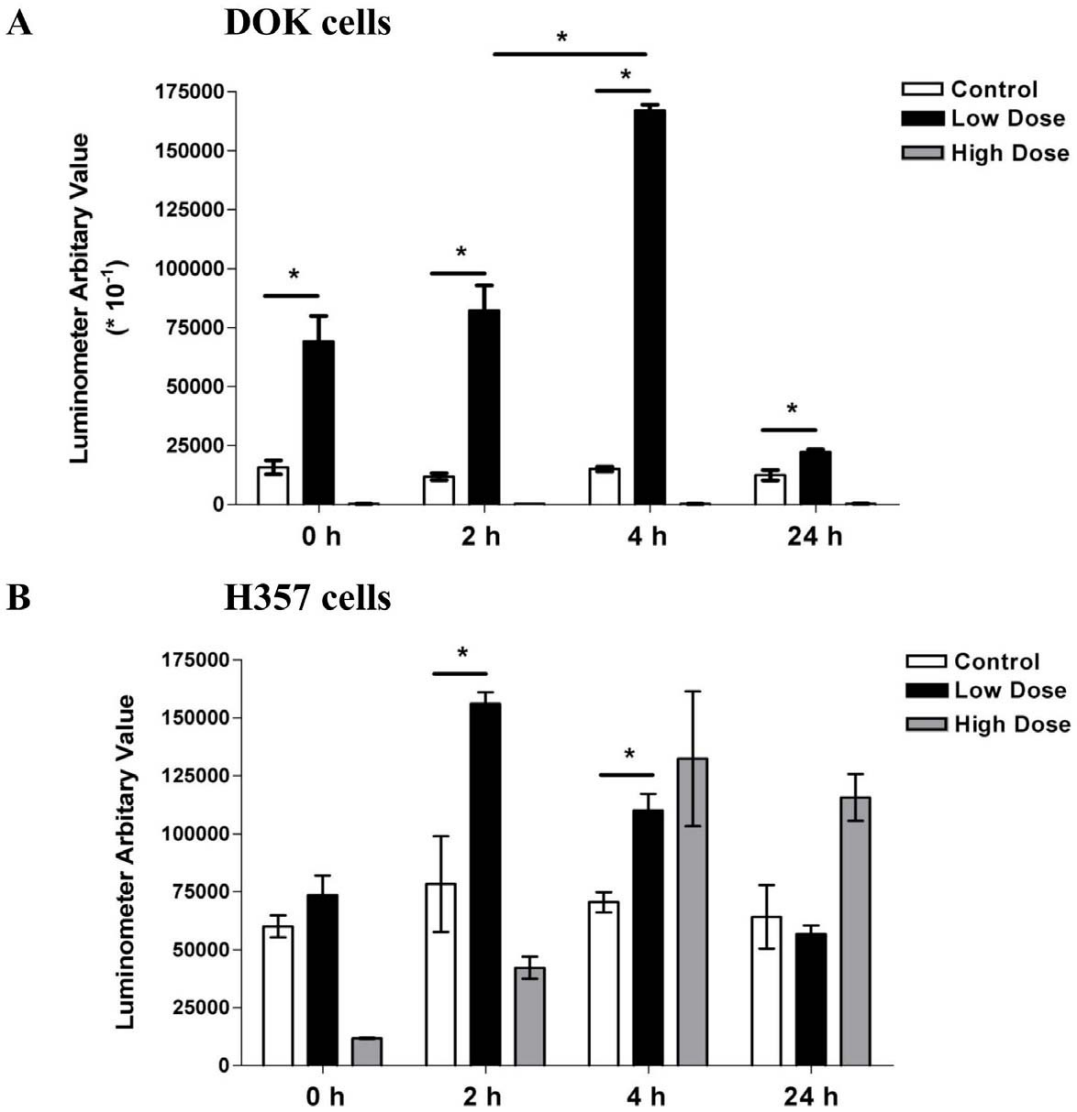
Caspase 3/7 Activity Assay. Caspase 3/7 activation in response to PDT was estimated at four different recovery time points post-PDT (0, 2, 4 and 24 hr) in DOK (**A**) and H357 cells (**B**) at both low (71.03 µM) and high (1136.50 µM) doses of erythrosine. For both doses, PDT irradiation was done with a fluence of 122.58 J/cm^2^. In order to bring the data sets of both DOK and H357 cells to the same scale, the luminometer arbitrary values for DOK cells were divided by 10. The values are means of three replicate determinations ± S.D. Statistical analysis was done *via* student's t-test with significance set at P<0.05 (* indicates statistical comparison as indicated by the lines).

Untreated (Control) DOK and H357 cells showed a constant, basal caspase 3/7 activity at all recovery time points. But after a low dose of erythrosine-PDT (71.03 µM–122.58 J/cm^2^), we observed an increase in the caspase 3/7 activity in both the cell lines (Figure 8A and 8B). The caspase 3/7 activity initially increased in the early recovery time points (Figure 8) and returned back to the control levels by 24 hr after treatment suggesting the occurrence of caspase-dependent apoptosis at early recovery time point culminating into secondary necrosis following PDT. This confirms the results seen with CLSM and SEM studies. Following high dose erythrosine-PDT (1136.5 µM–122.58 J/cm^2^), DOK cells showed caspase 3/7 activity below the control levels suggesting the occurrence of necrosis (Figure 8A). Again this confirms the results obtained with the CLSM and SEM studies. Contrary to this, the H357 cells showed an increase in caspase 3/7 activity following high PDT dose treatment suggesting the occurrence of apoptosis and a more limited necrotic response (Figure 8B). This limited necrotic response of H357 cells might go some way toward explaining the apparent resistance of these cells to erythrosine-PDT.

## Discussion

In common with other therapeutic strategies, PDT is constantly evolving and continues to be improved further [7], [30]. A defining feature of the development of PDT is the discovery of newer and more powerful photosensitizers [5], [14], [30]. To this end we have been investigating Erythrosine B as a potential photosensitizer for PDT-based treatment of oral malignancies.

The concentration of photosensitizer (PS) taken up by the cells is a vital parameter in cell killing efficacy [30]. Erythrosine uptake in DOK and H357 cells increased in an erythrosine concentration-dependent manner. In addition, the overall uptake was higher at 37°C than at 4°C suggesting that both active and passive uptake pathways are operating. Cells usually take up chemicals/drugs through either passive diffusion or through active transport [31] usually in association with various serum proteins such as lipoproteins, albumin and globulins [32] or by pinocytosis especially *via* bovine/calf serum albumin [33]. Lower temperatures affect lipid membrane structure [34] thereby preventing active transport of drugs into the cell [35]. Thus at 4°C, passive transport is usually predominant.

DOK and H357 cells showed a dose-dependent response to the PDT treatment. Maximum cell killing was observed at 1136.5 µM erythrosine and 122.58 J/cm^2^ irradiation fluence in DOK (∼80%) as well as H357 (∼60%) cells. It is apparent that DOK (pre-malignant) cells showed more susceptibility to erythrosine-based PDT than H357 (malignant) cells. This difference in susceptibility to PDT could be occurring for various reasons. One possible reason is the expression of telomerase which promotes cell survival by protecting cells from apoptosis [36], [37], [38]. Other protective mechanisms that might play a role in the resistance of H357 cells to erythrosine-based PDT include; (1) induction of drug-detoxifying mechanisms, (2) resistance to drug-induced apoptosis [39], (3) Mutational difference between DOK and H357 cells (p53 and Ha-ras gene is mutated in H357 cells) and (4) increased expression of anti-oxidants. Further, it was noted that for low PDT-doses, the standard deviation was large whereas for high PDT-doses the data spread was much lower. This variability in data might be explained by the fact that low PDT-stress resulting from low dosage has the ability of inducing several different responses in the treated cells *e.g.* apoptosis, repair, survival tactics and autophagy, while high PDT-stress results in induction of quick responses such as necrosis [21].

Erythrosine was found to be predominantly localizing in the mitochondria, for DOK as well as H357 cell types. We therefore attempted to find out whether erythrosine-based PDT causes any change in the mitochondrial trans-membrane potential (Δ**ψ_m_**). Our results showed that the H357 cells are far more resistant to changes in Δ**ψ_m_** when compared to the DOK cells. Dissipation of Δ**ψ_m_** in DOK cells was observed with both high and low doses of erythrosine which suggests it as a primary result of PDT-induced damage. However, there was less variation in the Δ**ψ_m_** of H357 cells where both the high and low doses produced a shift in potential only at the 24 hr recovery time point. This suggests that in the H357 cells there is a mechanism occurring at the cellular level which is capable of rescuing the cells from the effect of PDT. The precise mechanism by which depletion of Δ**ψ_m_** is causing cell death is unclear, but it has been reported that the dissipation of Δ**ψ_m_** will actually cause the release of cytochrome c and activate apoptosis [40], [41]. Other reports conclude that there is no relationship between Δ**ψ_m_** and release of cytochrome c along with apoptosis [42]. Nevertheless, our data suggests that the dissipation of Δ**ψ_m_** following PDT has significantly deleterious effects on cell survival. Interestingly, it has also been reported previously that the dissipation in Δ**ψ_m_** actually enhances the permeability of lysosomal membranes, resulting in the release of lysosomal proteins and subsequent activation of cell death pathways accompanied by autophagy [43].

Cell death morphology analysis using CLSM and SEM, demonstrated the occurrence of apoptosis following low dose PDT and necrosis with high dose in both the cell lines. In H357 cells high dose PDT induced apoptosis in addition to necrosis. It has been reported previously by Castano *et al* that higher doses of PDT result predominantly in necrosis depending upon the cell line and the low dose in PDT results in apoptosis [44]. Kessel *et al* have also reported the higher incidence of apoptosis during low dose PDT and inhibition of apoptosis at high dose with aluminum phthalocyanine chloride and two other porphycene sensitizers [45].

This is confirmed by the results of the caspase 3/7 activity analysis presented in this study which suggested the occurrence of apoptosis following low dose PDT in both cell lines during the initial recovery time points. An increase in caspase 3/7 activity was also seen using high dose PDT in H357 cells suggesting the occurrence of apoptosis alongside necrosis in this cell line.

As expected, high dose PDT of DOK cells resulted in caspase 3/7 activity below control levels which is supported by the SEM/CLSM data and suggests that necrosis is the main mechanism of cell death at this dose. This occurrence of extensive necrosis in the DOK cells is probably the main reason for the higher death percentage in DOK cells when compared to the H357 cells. Kessel *et al* (1997) and Shah *et al* (1996) have reported that extensive necrosis is promoted when there is an increased photo-inactivation of caspases and inhibition of apoptosis [45], [46]. The result of this study has shown us that the H357 are far more resistant than the DOK cells.

To conclude, we have found that erythrosine is an effective photosensitizer for PDT treatment of both malignant and pre-malignant oral cell lines. In fact, to the best of our knowledge, this is the first instance of erythrosine-based PDT being applied to kill cancer cells. However, the malignant H357 cell line was more resistant to PDT which may be related to a lower necrotic response following PDT. Further studies are needed to elucidate the precise mechanisms of cell killing at a molecular level. Moreover it would be also vital in the near future, to analyze if erythrosine offers better pharmacokinetics and physio-chemical properties than existing photosensitizers.

## Materials and Methods

### Cell Culture Conditions

The two oral epithelial cell lines (DOK and H357) were purchased from the European Collection of Cell Cultures (ECACC). DOK (pre-malignant; ECACC No. 94122104) [47] and H357 (malignant; ECACC No. 06092004) [48] cells were derived from dysplastic oral keratinocytes and squamous cell carcinoma (SCC) of the tongue respectively. Both cell lines were cultured in Dulbecco's modified Eagle's medium (DMEM) (Sigma-Aldrich, UK) supplemented with 10% Fetal Calf Serum (Sigma-Aldrich, UK), 50 U/ml of Penicillin-Streptomycin (Sigma-Aldrich, UK) and 5 µg/ml of Hydrocortisone (Sigma-Aldrich, UK) and maintained at 37°C with 5% CO_2_ atmosphere.

### Preparation of Erythrosine

Erythrosine B sodium salt (Sigma-Aldrich, UK) was dissolved in DMEM to give a final concentration of 1 mg/ml. This solution was then sterilized using a 0.2 µm filter and serially diluted to produce photosensitizer concentrations of 0.5 mg/ml, 0.25 mg/ml, 0.125 mg/ml and 0.0625 mg/ml for use in the following studies.

### Light Source:-

A 400W Tungsten filament lamp with a fluence rate of 22.7 mWcm^−2^ in the 500–550 nm wavelength range was used for illumination of samples. A water tank was used to remove infra-red. Samples were placed under the water tank at a distance of 30 cm from the lamp during irradiation. The irradiance was calculated under the water tank at a distance of 30 cm from the lamp (treatment location). Throughout this study, control samples are cells incubated with erythrosine B but not irradiated (*i.e.* untreated samples).

### Erythrosine Uptake Analysis

DOK and H357 cells were seeded in 6-well plates (10^4^ cells/well) and returned to the incubator overnight. The cells were then incubated with erythrosine at concentrations of 0.0625 mg/ml (71.03 µM), 0.125 mg/ml (142.06 µM), 0.25 mg/ml (284.13 µM), 0.5 mg/ml (568.27 µM) and 1 mg/ml (1136.5 µM) at 37°C or at 4°C for 30 min. The cells were then washed (×3) with Dulbecco's Phosphate Buffer Saline (PBS), incubated with 0.1 M NaOH (1 ml, for 1–2 min) and harvested using a cell scraper. The samples were then diluted (1∶10) with 0.1 M NaOH followed by determination of fluorescence intensity using an excitation (550 nm) and emission (540 nm) wavelength in the Microplate Reader (Dynex Technologies Ltd, UK). Erythrosine concentration in each sample was determined by comparison of the sample's fluorescence with that of a standard erythrosine solution in 0.1 M NaOH. The amount of protein in each sample was also determined using the BCA (bicinchoninic acid) Protein Assay Kit (Pierce, UK), as per the manufacturer's instructions. All erythrosine uptake experiments were carried out in quadruplicate for each treated and control sample. Erythrosine uptake was expressed as µg erythrosine/mg cell protein.

### PDT based Cell Killing

DOK and H357 cells were plated in 96-well plates (5×10^3^ cells/well) 24 hours before the treatment. Duplicate wells were incubated with erythrosine concentrations of 0.0625 mg/ml (71.03 µM), 0.125 mg/ml (142.06 µM), 0.25 mg/ml (284.13 µM), 0.5 mg/ml (568.27 µM) and 1 mg/ml (1136.5 µM) for 30 min. Cell monolayers were then washed (×3 with PBS) and subjected to PDT using 0, 30 (40.86 J/cm^2^), 60 (81.72 J/cm^2^) and 90 (122.58 J/cm^2^) min irradiation times (fluences). After irradiation, plates were incubated at 37°C for 12 hr before quantification of cell viability using the CellTiter 96®AQ_ueous_ One Solution Cell Proliferation Assay (Promega, USA) as per the manufacturer's instructions. Formazan formed by viable cells was quantified by reading the plates at 490 nm using a Microplate Reader (Dynex Technologies Ltd., UK). Cell kill following PDT were converted into percentage cell kill using the following formulae:
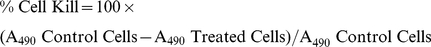



### Sub-cellular Localization Analysis by CLSM

DOK and H357 cells were seeded in Sterilin Petri-dish. Following attachment they were incubated with a concentration of 1136.5 µM erythrosine at 37°C for 30 min in the dark. For organelle localization comparison, cells were either co-incubated with 100 nM LysoTracker Green (Invitrogen) or 100 nM MitoTracker Green (Invitrogen). Samples were then observed directly using a Leica TCS SP2 Confocal Laser Scanning Microscope (CLSM). All the images were analyzed *via* ImageJ software (W. S. Rasbaud, Image J, NIH, Bethesda, MD, http://rsb.info.nih.gov/ij/). Co-localization of either LysoTracker or MitoTracker with erythrosine was (statistically) evaluated through the agency of co-localization frequency scatter plots, Pearson's coefficient and Mander's coefficient as explained elsewhere [29].

### Mitochondrial Trans-Membrane Potential (ΔΨ_m_) Analysis via Flow Cytometry

DOK and H357 cells were seeded in 21.5 cm^2^ cell culture plates as described above. Following incubation with erythrosine concentrations of 0.0625 mg/ml (71.03 µM) and 1 mg/ml (1136.5 µM) for 30 min, the cells were irradiated for 90 min (122.58 J/cm^2^). Cells were then trypsinised at three different recovery time-points (0, 2 and 24 h) after irradiation followed by cell pelleting and washing with PBS. The cell pellet was then resuspended in PBS containing 5 µg/ml of Rhodamine B followed by incubation for 30 min at 37°C. Subsequently, the cells were analyzed using the FACS Calibur Flow Cytometry (BD Biosciences, USA). The results were processed using *CellQuest Software* (BD Biosciences, USA). For each sample, 10,000 events were collected. The results generated by CellQuest software were processed using the software, *WinMDI v.2.9* (http://facs.scripps.edu/software.html). Here, the intensity of rhodamine B fluorescence was taken as direct indication of overall mitochondrial membrane potential (ΔΨ_m_) for a particular cell population. All the calibrations of the flow cytometer were set-up by Dr. Gareth Howell (Research Fellow, Institute of Molecular and Cellular Biology, Faculty of Biological Sciences, University of Leeds, UK).

### Cell Death Morphology Analysis using Scanning Electron Microscopy (SEM)

DOK and H357 cells were prepared as stated above in 21.5 cm^2^ cell culture plates for two different erythrosine concentrations −0.0625 mg/ml (71.03 µM) and 1 mg/ml (1136.5 µM). Following incubation with erythrosine, the cells were irradiated for 90 min (122.58 J/cm^2^) followed by fixation for 1 hr using 2.5% gluteraldehyde in PBS (pH 7.4) at three different recovery time-points (0,2 and 24 h) after irradiation. Subsequently, the cells were washed with PBS (3×) to ensure complete gluteraldehyde removal. Samples were then dehydrated through a graded ethyl alcohol series. Following dehydration, the plates were cut into suitable pieces, fixed to aluminum stubs and sputter-coated with gold using an Automatic Sputter Coater (Agar Scientific, UK). The samples were then visualized directly using a Hitachi VP-SEM S-3400N Scanning Electron Microscope.

### Cell Death Morphology Analysis using CLSM

DOK and H357 cells were prepared as stated above in 21.5 cm^2^ cell culture plates for two different erythrosine concentrations −0.0625 mg/ml (71.03 µM) and 1 mg/ml (1136.5 µM) and subjected to PDT −90 min irradiation (122.58 J/cm^2^). Following PDT, the cells were stained using SYT09 stain (green fluorescence; excitation λ- 420 nm, emission λ-580 nm) which stains the nucleic acid in both live/dead cells and Propidium Iodide (PI) (red fluorescence; excitation λ- 420 nm, emission λ-580 nm) which stains the nucleic acids of dead cells alone; at recovery time points of 2, 4 and 24 h following irradiation. Cells were then observed immediately using a Leica TCS SP2 CLSM.

### Caspase Activity Assay

Samples were prepared as stated above in 96-well plate for two different erythrosine concentrations −0.0625 mg/ml (71.03 µM) and 1 mg/ml (1136.5 µM) and subjected to PDT −90 min irradiation (122.58 J/cm^2^). Following PDT, the Caspase activity assay was performed using the Caspase-Glo3/7 assay (Promega, Madison, USA) kit at four different recovery time point (0, 2, 4 and 24 h) as per the manufacturer's instruction. Then the plates were observed for the luminescence signal in the luminometer (BMG Labtech Fluostar Optima, Germany). Calibrations of the luminometer were performed by Dr. Carsten Zoothner (Technician, Institute of Molecular and Cellular Biology, University of Leeds, UK).

## References

[1] British Dental Association (2000). Opportunistic Oral Cancer Screening: A management strategy for Dental Practice..

[2] Argiris A, Eng C, Brockstein B, Masters G (2004). Epidemiology, staging and screening of head and neck cancer.. Head and Neck Cancer.

[3] Parkin DM, Bray F, Ferlay J, Pisani P (2005). Global cancer statistics, 2002.. CA Cancer J Clin.

[4] Konopka K, Goslinski T (2007). Photodynamic therapy in dentistry.. J Dent Res.

[5] Garg AD, Krysko DV, Vandenabeele P, Agostinis P (2011). DAMPs and PDT-mediated photo-oxidative stress: exploring the unknown.. Photochem Photobiol Sci.

[6] Garg AD, Nowis D, Golab J, Agostinis P (2010). Photodynamic therapy: illuminating the road from cell death towards anti-tumour immunity.. Apoptosis.

[7] Agostinis P, Berg K, Cengel KA, Foster TH, Girotti AW (2011). Photodynamic therapy of cancer: An update.. CA Cancer J Clin.

[8] Garg AD, Krysko DV, Vandenabeele P, Agostinis P (2012). Hypericin-based photodynamic therapy induces surface exposure of damage-associated molecular patterns like HSP70 and calreticulin.. Cancer Immunol Immunother.

[9] Garg AD, Krysko DV, Verfaillie T, Kaczmarek A, Ferreira GB (2012). A novel pathway combining calreticulin exposure and ATP secretion in immunogenic cancer cell death.. EMBO J.

[10] Dougherty TJ, Gomer CJ, Henderson BW, Jori G, Kessel D (1998). Photodynamic therapy.. J Natl Cancer Inst.

[11] Estevez JP, Ascencio M, Colin P, Farine MO, Collinet P (2010). Continuous or fractionated photodynamic therapy? Comparison of three PDT schemes for ovarian peritoneal micrometastasis treatment in a rat model.. Photodiagnosis and Photodynamic Therapy.

[12] Usuda J, Tsunoda Y, Ichinose S, Ishizumi T, Ohtani K (2010). Breast cancer resistant protein (BCRP) is a molecular determinant of the outcome of photodynamic therapy (PDT) for centrally located early lung cancer.. Lung Cancer.

[13] Kobayashi W, Liu Q, Nakagawa H, Sakaki H, Teh B (2006). Photodynamic therapy with mono-l-aspartyl chlorin e6 can cause necrosis of squamous cell carcinoma of tongue: Experimental study on an animal model of nude mouse.. Oral oncology.

[14] Agostinis P, Buytaert E, Breyssens H, Hendrickx N (2004). Regulatory pathways in photodynamic therapy induced apoptosis.. Photochem Photobiol Sci.

[15] Josefsen LB, Boyle RW (2008). Photodynamic therapy: novel third-generation photosensitizers one step closer?. Br J Pharmacol.

[16] Allaker RP, Douglas CW (2009). Novel anti-microbial therapies for dental plaque-related diseases.. Int J Antimicrob Agents.

[17] Wood S, Metcalf D, Devine D, Robinson C (2006). Erythrosine is a potential photosensitizer for the photodynamic therapy of oral plaque biofilms.. J Antimicrob Chemother.

[18] Gandin E, Lion Y, Van de Vorst A (1979). Production of radicals by singlet oxygen reaction: an E.S.R. study [proceedings].. Arch Int Physiol Biochim.

[19] Allison RR, Downie GH, Cuenca R, Hu X-H, Childs CJH (2004). Photosensitizers in clinical PDT.. Photodiagnosis and Photodynamic Therapy.

[20] Nagel W, Somieski P, Katz U (2001). Selective inhibition of Cl(−) conductance in toad skin by blockers of Cl(−) channels and transporters.. Am J Physiol Cell Physiol.

[21] Plaetzer K, Kiesslich T, Verwanger T, Krammer B (2003). The Modes of Cell Death Induced by PDT: An Overview.. Med Laser Appl.

[22] He YY, Council SE, Feng L, Bonini MG, Chignell CF (2008). Spatial distribution of protein damage by singlet oxygen in keratinocytes.. Photochem Photobiol.

[23] Moan J, Berg K (1991). The photodegradation of porphyrins in cells can be used to estimate the lifetime of singlet oxygen.. Photochem Photobiol.

[24] Moor ACE, Ortel B, Hasan T, Patrice T (2003). Mechanisms of photodynamic therapy.. Photodynamic Therapy.

[25] Buytaert E, Dewaele M, Agostinis P (2007). Molecular effectors of multiple cell death pathways initiated by photodynamic therapy.. Biochim Biophys Acta.

[26] Garg AD, Nowis D, Golab J, Vandenabeele P, Krysko DV (2010). Immunogenic cell death, DAMPs and anticancer therapeutics: an emerging amalgamation.. Biochim Biophys Acta.

[27] Hengartner MO (2000). The biochemistry of apoptosis.. Nature.

[28] Green DR, Reed JC (1998). Mitochondria and apoptosis.. Science.

[29] Bolte S, Cordelieres FP (2006). A guided tour into subcellular colocalization analysis in light microscopy.. J Microsc.

[30] Moan J, Peng Q, Patrice T (2003). An outline of the history of PDT.. Photodynamic Therapy.

[31] Alberts B, Johnson A, Lewis J, Raff M, Roberts K (2002). Molecular Biology of the Cell.

[32] Sharman WM, van Lier JE, Allen CM (2004). Targeted photodynamic therapy via receptor mediated delivery systems.. Adv Drug Deliv Rev.

[33] Siboni G, Weitman H, Freeman D, Mazur Y, Malik Z (2002). The correlation between hydrophilicity of hypericins and helianthrone: internalization mechanisms, subcellular distribution and photodynamic action in colon carcinoma cells.. Photochem Photobiol Sci.

[34] Vazifeh D, Bryskier A, Labro MT (1999). Mechanism underlying levofloxacin uptake by human polymorphonuclear neutrophils.. Antimicrob Agents Chemother.

[35] Friberg EG, Cunderlikova B, Pettersen EO, Moan J (2003). pH effects on the cellular uptake of four photosensitizing drugs evaluated for use in photodynamic therapy of cancer.. Cancer Lett.

[36] Geserick C, Blasco MA (2006). Novel roles for telomerase in aging.. Mechanisms of Ageing and Development.

[37] Loebinger MR, Sage EK, Davies D, Janes SM (2010). TRAIL-expressing mesenchymal stem cells kill the putative cancer stem cell population.. Br J Cancer.

[38] Indran IR, Hande MP, Pervaiz S (2011). hTERT Overexpression Alleviates Intracellular ROS Production, Improves Mitochondrial Function, and Inhibits ROS-Mediated Apoptosis in Cancer Cells.. Cancer Research.

[39] Gottesman MM (2002). Mechanisms of cancer drug resistance.. Annu Rev Med.

[40] Schuler M, Bossy-Wetzel E, Goldstein JC, Fitzgerald P, Green DR (2000). p53 Induces Apoptosis by Caspase Activation through Mitochondrial Cytochrome c Release.. Journal of Biological Chemistry.

[41] Chaloupka R, Petit PX, Israël N, Sureau F (1999). Over-expression of Bcl-2 does not protect cells from hypericin photo-induced mitochondrial membrane depolarization, but delays subsequent events in the apoptotic pathway.. FEBS Letters.

[42] Chiu SM, Oleinick NL (2001). Dissociation of mitochondrial depolarization from cytochrome c release during apoptosis induced by photodynamic therapy.. Br J Cancer.

[43] Kim R, Emi M, Tanabe K, Murakami S, Uchida Y (2006). Regulation and interplay of apoptotic and non-apoptotic cell death.. The Journal of Pathology.

[44] Castano AP, Demidova TN, Hamblin MR (2005). Mechanisms in photodynamic therapy: part two–cellular signaling, cell metabolism and modes of cell death.. Photodiagnosis and Photodynamic Therapy.

[45] Luo Y, Kessel D (1997). Initiation of Apoptosis versus Necrosis by Photodynamic Therapy with Chloroaluminum Phthalocyanine.. Photochemistry and Photobiology.

[46] Shah GM, Shah RG, Poirier GG (1996). Different Cleavage Pattern for Poly(ADP-Ribose) Polymerase during Necrosis and Apoptosis in HL-60 Cells.. Biochemical and Biophysical Research Communications.

[47] Chang SE, Foster S, Betts D, Marnock WE (1992). DOK, a cell line established from human dysplastic oral mucosa, shows a partially transformed non-malignant phenotype.. International Journal of Cancer.

[48] Prime SS, Matthews JB, Patel V, Game SM, Donnelly M (1994). TGF-β receptor regulation mediates the response to exogenous ligand but is independent of the degree of cellular differentiation in human oral keratinocytes.. International Journal of Cancer.

